# The minimally invasive surgical local osteo-enhancement procedure (LOEP) to deliver a resorbable, tri-phasic calcium-based implant material to address bone loss and strengthen the proximal femur

**DOI:** 10.3389/fsurg.2025.1661501

**Published:** 2025-12-19

**Authors:** H. Goost, J. De Schepper, J. D. Rölfing, H. Aguado, J. Howe, B. Huber

**Affiliations:** 1 Department of Orthopaedics and Trauma Surgery, Wermelskirchen Hospital, Wermelskirchen, Germany; 2 Department of Orthopaedics, Vitaz Campus Sint-Niklaas, Sint-Niklaas, Belgium; 3 Department of Orthopaedics and Traumatology, Aarhus University Hospital, Aarhus, Denmark; 4 Department of Orthopedics and Trauma, University Clinical Hospital of Valladolid, Valladolid, Spain; 5 Department of Medical Affairs, AgNovos Healthcare, Rockville, MD, United States

**Keywords:** osteoporosis, augmentation, osteo-enhancement, biomechanics, femoroplasty, hip fracture, prevention, LOEP

## Abstract

With global population growth and advancing age, fragility fractures present a major healthcare challenge that current approaches have not resolved. Although pharmacological agents have been shown to reduce fragility fracture risk, there remain unmet needs in clinical care, especially for patients at imminent risk of hip fracture, given the delay between treatment initiation and observed protective effect. This gap suggests a need for novel approaches, including hip procedures that strengthen bone locally and quickly. The purpose of this report is to describe a procedural technique that has been shown in ex vivo and clinical studies to rapidly enhance proximal femur biomechanical properties and leads to new bone formation. Local osteo-enhancement procedure (LOEP) is a minimally invasive surgical procedure to address bone voids in the pelvis and extremities, including in the proximal femur, due to trauma and disease such as osteoporosis. After surgical preparation of voids within the femoral neck and intertrochanteric regions of the proximal femur, a resorbable, triphasic, calcium-based implant material, AGN1, is delivered to that site. Clinical studies demonstrate consistent implant material resorption, concurrent replacement of the material with bone, and a significant, durable increase in areal bone mineral density (aBMD). The procedural technique has been studied as a standalone procedure and as concomitant surgery taking place in the same operative session as the surgical treatment of a contralateral index hip fragility fracture. In the study of concomitant use, LOEP was reported not to disrupt the standard of care for mobilization and rehabilitation for hip fracture. The clinical studies completed suggest that the procedure demonstrates an acceptable safety profile and has the potential to reduce the incidence of hip fragility fractures.

## Introduction

Osteoporosis and osteopenia are systemic skeletal diseases characterized by low bone mass and microarchitectural deterioration of bone tissue. They manifest as increased cortical porosity and reduced trabecular bone volume, leading to decreased bone strength, and increased risk of fracture. The degree of bone loss and strength loss depends on multiple factors. However, Verhulp et al., reported that the resistance to normal hip-joint loading of an osteoporotic femur is 59% of that of a normal femur (1). According to the World Health Organization, osteoporosis is defined by a bone mineral density (BMD) T-score ≤−2.5, while osteopenia refers to a T-score between −1.0 and −2.5 (2). The global prevalence of osteoporosis and osteopenia has been estimated to be ∼20% and 40%, respectively (3). It is estimated that one in three women and one in five men over the age of 50 will experience an osteoporotic fracture during their lifetime (4). The risk of fracture rises exponentially with decreasing BMD, as each standard deviation reduction in BMD approximately doubles fracture risk (5).

Fragility fractures result from low-energy trauma, such as a fall from a standing height or less. These fractures represent the most severe consequences of osteoporosis. It is estimated that 30% of individuals aged 65 years or older will experience at least one fall per year (6), highlighting the high exposure of this population to the initiating event that, combined with reduced bone strength, can lead to fractures (6).

Common fragility fracture sites include the hip, distal radius, proximal humerus, spine and the pelvic ring, which together account for most osteoporotic fractures seen in clinical practice (7). Among fragility fractures, hip fractures are associated with the highest mortality and socioeconomic burden for patients, families and healthcare systems (8–10). The one-year mortality after a hip fracture ranges from 20% to 36%, largely due to postoperative complications, immobility and comorbidities in the elderly population (8, 11–14). Fractures also cause chronic pain and disability. Approximately 20%–50% of fracture patients require long-term nursing home care and suffer from decreased quality of life (4). Although hip fractures account for only 14% of all fragility fractures, they account for roughly 72% of total fracture-related costs (10). The global burden of hip fractures has increased substantially—from an estimated 1.3 million cases in 1990 to more than 14 million in 2019—and is projected to double again by 2050 (15, 16). This growing burden has been described as a “crisis” by multiple expert groups, underscoring the urgent need for novel strategies to reduce the risk of fragility fractures (13, 17–19).

Current treatment guidelines for osteoporosis are developed by multidisciplinary teams comprising geriatricians, endocrinologists, and orthopedic surgeons, and typically combine patient education, nutrition, exercise, and pharmacological therapy. Non-pharmacological interventions target modifiable risk factors such as diet modification, smoking cessation, excessive alcohol avoidance, and regular weight-and muscle-strengthening exercise (20–22). While regular weight-bearing activities (e.g., walking, stair climbing) and progressive resistance or balance training (e.g., Tai Chi, strength exercises) have been shown to improve bone density and reduce risk of falling, there is a lack of evidence showing that they reduce fracture risk (18). Pharmacologic treatments aim to decrease bone loss or stimulate bone formation, thereby increasing bone mass and bone strength (22). Anti-resorptive agents such as bisphosphonates or denosumab inhibit osteoclast-mediated bone loss, anabolic agents such as teriparatide stimulate osteoblast activity; romosozumab does both (23). While these systemic therapies are effective at reducing overall fracture incidence, their protective effect at the hip has been shown to be limited to approximately 50% risk reduction (24–34). Moreover, adherence rates fall below 50% after one year due to side effects, complex dosing regimens and the long time—often 12–18 months—required to achieve measurable therapeutic effect at the hip (24–36). Consequently, there remains a clear need for novel, targeted strategies to further reduce risk.

Preventative strategies to reduce hip fracture risk have long been explored. External hip protectors have been studied but suffer from poor comfort and compliance (37). Surgical strategies aimed at mechanically reinforcing the proximal femur using permanent materials, such as metal implants or non-resorbable cements, have also been investigated, but these approaches are limited by their creating a mismatch in biomechanical properties vs. native bone, increasing the risk for long-term complications (38, 39).

The local osteo-enhancement procedure (LOEP) was developed as a percutaneous, surgical approach to locally strengthen osteoporotic bone by addressing bone voids including those in the proximal femur caused by trauma and bone diseases such as osteoporosis (40). LOEP involves the injection of a bioresorbable, calcium-salt-based material that hardens *in situ* with an exothermic reaction that does not exceed normal body temperature, improving the mechanical stability of the proximal femur (40). Pre-clinical and clinical studies have demonstrated its safety, capacity to increase local bone strength and support new bone formation (41–45). This manuscript presents a detailed description of the surgical technique for this treatment.

## Methods

### Setting

**Figure 1:**
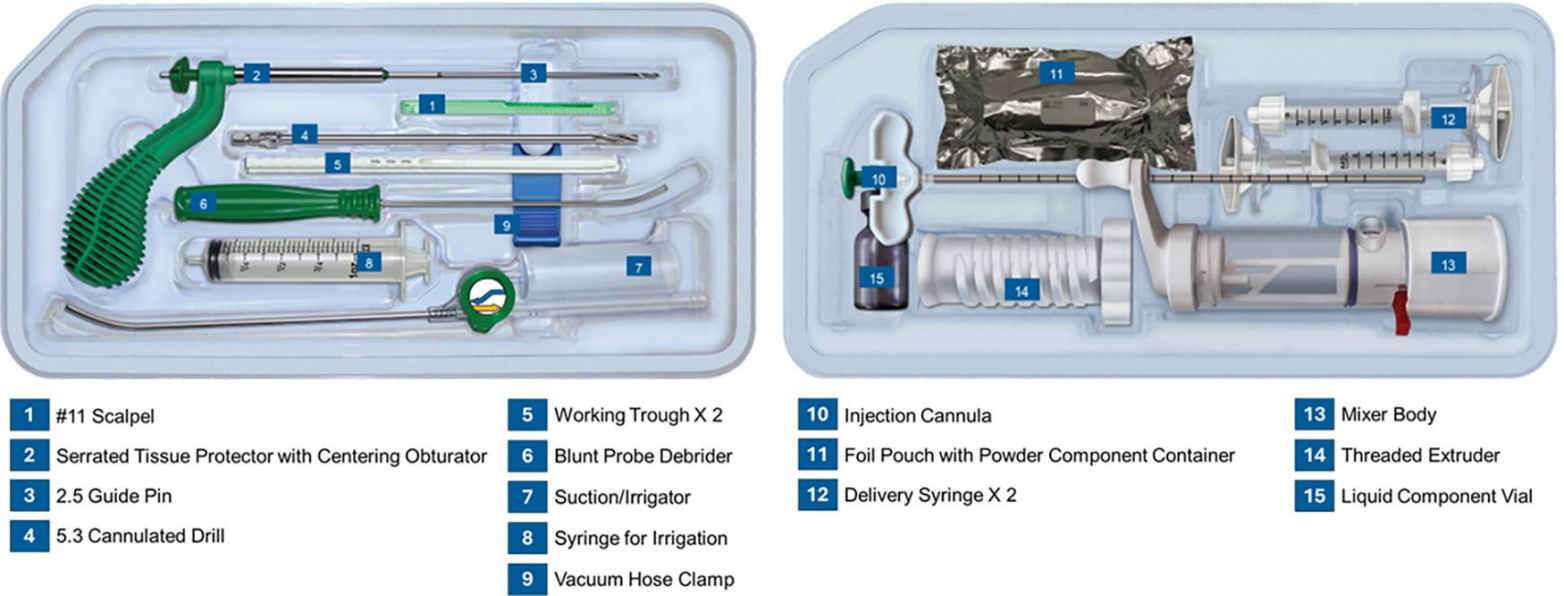
Illustration of the instruments and implant material components provided in the AGN1 LOEP kit. The instruments are used to access and prepare the site for treatment as well as to mix and inject the AGN1 implant material.

LOEP is being used as a locally, targeted adjunct to standard osteoporosis care and has been studied in prospective and retrospective clinical studies in the US, Europe, and Asia. In Europe, the LOEP kit is CE-mark registered and permitted for use as indicated even outside of clinical studies according to national regulations (Figure 1—AGN1 LOEP Kit Components). LOEP is being performed as a standalone unilateral and bilateral procedure, and, as appropriate, concomitantly with contralateral index hip fracture surgery under the primary surgeon's discretion and institutional policy (42, 43). Procedures are typically performed on a fracture table with biplanar intraoperative fluoroscopic guidance. Anesthesia (i.e., local with sedation, spinal or general) is selected based on patient characteristics and input from the anesthesiologist. Typical skin-skin time is under 30 min with minimal blood loss (42).

### Indication and contraindications

#### Indication

The AGN1 resorbable implant material contained in the AGN1 LOEP Kit is intended to form new bone in voids in the skeletal system. It is intended to be injected into surgically prepared sites in the pelvis and extremities, where it cures *in situ*, and is resorbed and replaced with new bone. AGN1 is appropriate for use in voids not intrinsic to the structural stability of the skeletal system.

#### Contraindications

The AGN1 resorbable implant material is contraindicated where the device is used in articulating surfaces, or for structural support in lieu of hardware in load-bearing bone.

Conditions that represent relative contraindications include:

• Severe vascular or neurological disease
• Uncontrolled diabetes
• Severe degenerative bone disease that compromises the body's ability to replace the implant material with bone
• Closed voids/gaps that are structurally capable of over-pressurization during injection
• Pregnancy
• Uncooperative patients who will not or cannot follow postoperative instructions, including individuals who abuse drugs and/or alcohol
• Pre-existing calcium metabolism disorder (e.g,. hypercalcaemia)
• Renal compromised patients
• Patients with a history of or active tuberculous spondylitis

The AGN1 LOEP kit is contraindicated for use in spinal, cranial and craniomaxillofacial reconstructions applications (46).

### Equipment—instrumentation

The sterile, single-use LOEP kit includes: serrated tissue protector, centering obturator, 2.5 mm guide pin, 5.3 mm cannulated drill (to enlarge entry portal—no separate entry reamer is used), working trough, curved blunt probe debrider, suction/irrigator, hose clamp, injection cannula, mixing accessories (mixer body/threaded extruder) (Figure 1—AGN1 LOEP Kit Components).

### Material

Two powder components are mixed with a neutralized glycolic-acid aqueous solution to form the bioresorbable, AGN1 implant material comprised of calcium sulfate (CSD), calcium phosphate, and *β*-tricalcium phosphate (*β*-TCP). Once mixed, AGN1 is an injectable and radiopaque paste that hardens *in situ*. The cured material provides an osteoconductive scaffold for bone formation (43).

### Surgical technique

#### Patient positioning and imaging

**Figure 2:**
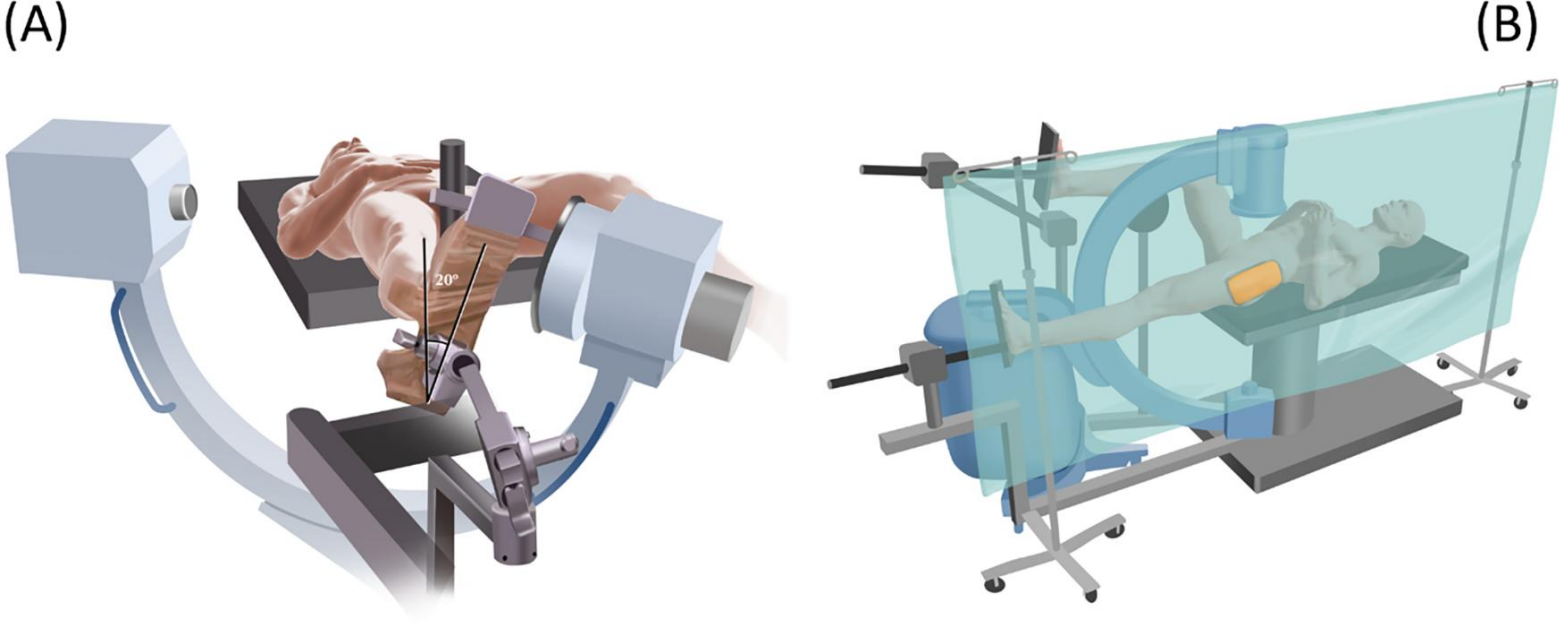
The left image **(A)** displays the patient in the supine position on the fracture table with internal rotation of the operative leg by approximately 20°. The right image **(B)** displays the setup after preparation, draping, and positioning for a fluoroscopy-guided AP image.

The patient is positioned supine on a fracture table; the non-operative lower extremity is flexed and in an abducted position to allow C-arm passage between the legs (Figure 2—patient positioning). The foot of the operative leg is placed in a table-attached boot, with only enough traction to stabilize the leg without distracting the hip, and the operative leg is internally rotated to align femoral neck anteversion parallel to the floor. High-quality AP and lateral fluoroscopic images must be obtained and confirmed before prepping and draping. If adequate views cannot be achieved, reposition and re-acquire images. Prep and draping should only occur once image acquisition and confirmation are complete. If proper fluoroscopic images are not obtained in both planes before preparation and draping, there is a potential for inadequate visualization and poor placement of the skin incision, guide pin, and drill. The ipsilateral arm is strapped across the body. If the patient is already on a fracture table for hip fracture repair, the surgeon will re-drape the contralateral hip and redirect the fluoroscopy for AP and lateral images to facilitate LOEP treatment.

#### Skin marking

**Figure 3:**
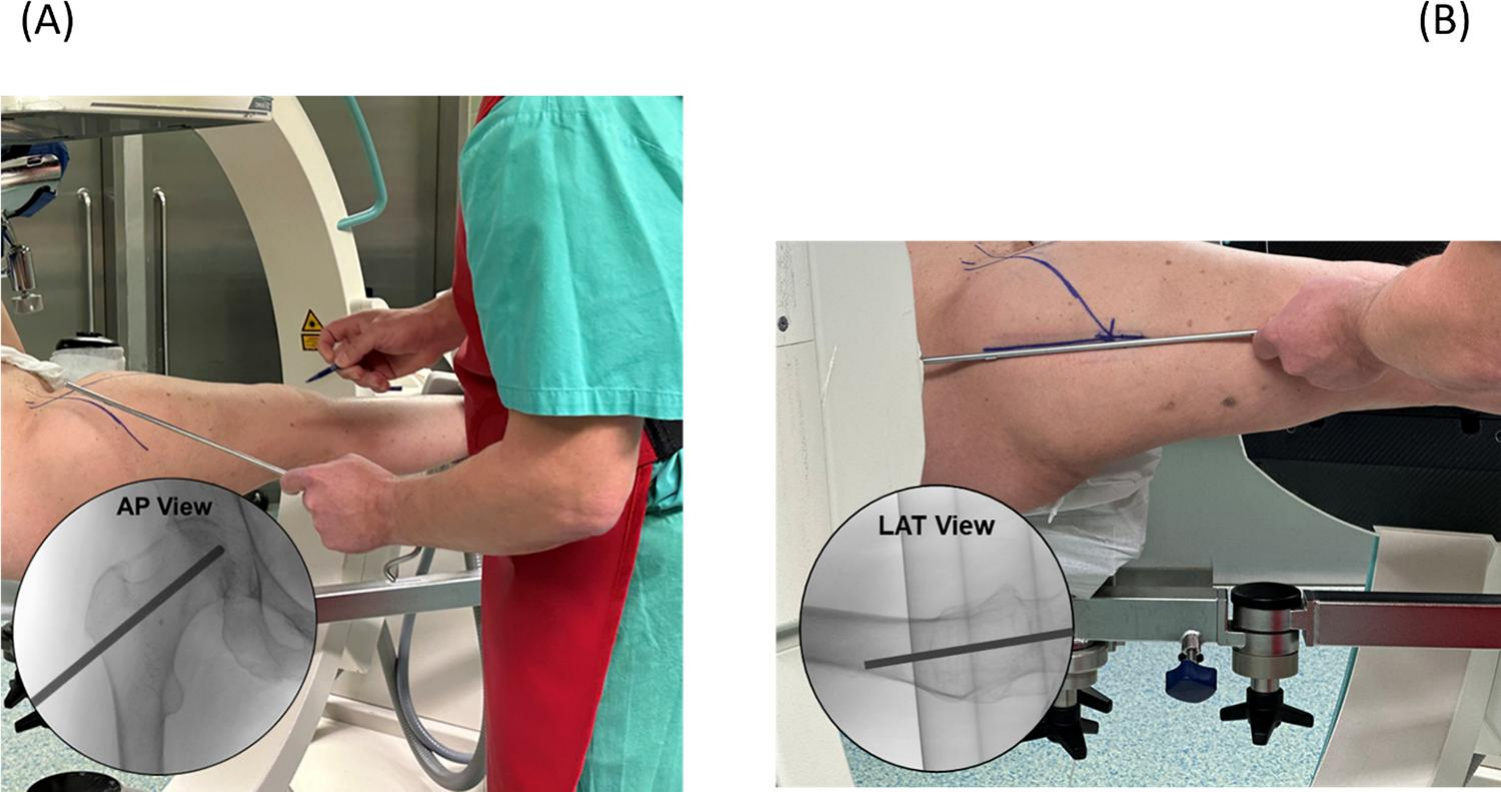
The left image **(A)** displays the patient in the supine position on the fracture table. A metallic, non-sterile instrument is being used to identify and mark the central trajectory in the AP plane, and the right image **(B)** marks the central position in the lateral plane. Both marks are prepared before prep and drape.

A non-sterile K-wire and fluoroscopy are used to mark the center-neck trajectory on AP and lateral images (Figure 3—skin marking). The lateral femur is prepped and draped in the usual sterile fashion. The skin mark and incision should be collinear with the lateral cortical entry site which is at or proximal to the most superior portion of the lesser trochanter.

#### Incision and access to the proximal femur

**Figure 4:**
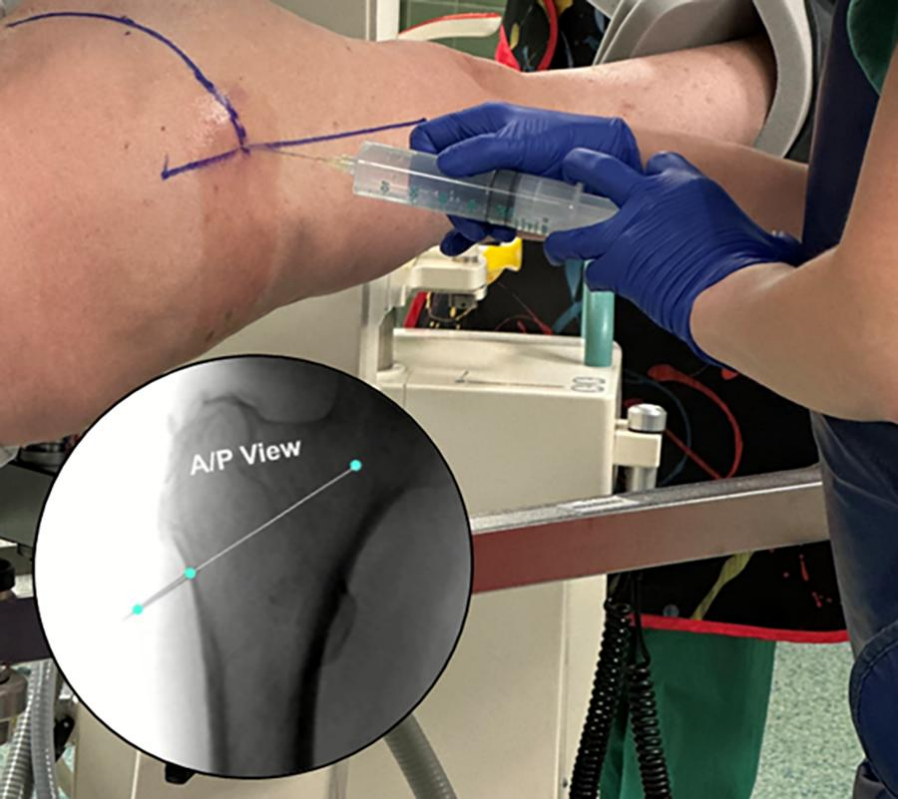
A local anesthesia injection is performed after prep and drape to localize and confirm the proper skin incision that is colinear with the projected lateral cortical entry. The fluoroscopic image confirms needle entry through the skin to the lateral femur, above the level of the most proximal portion of the lesser trochanter.

**Figure 5:**
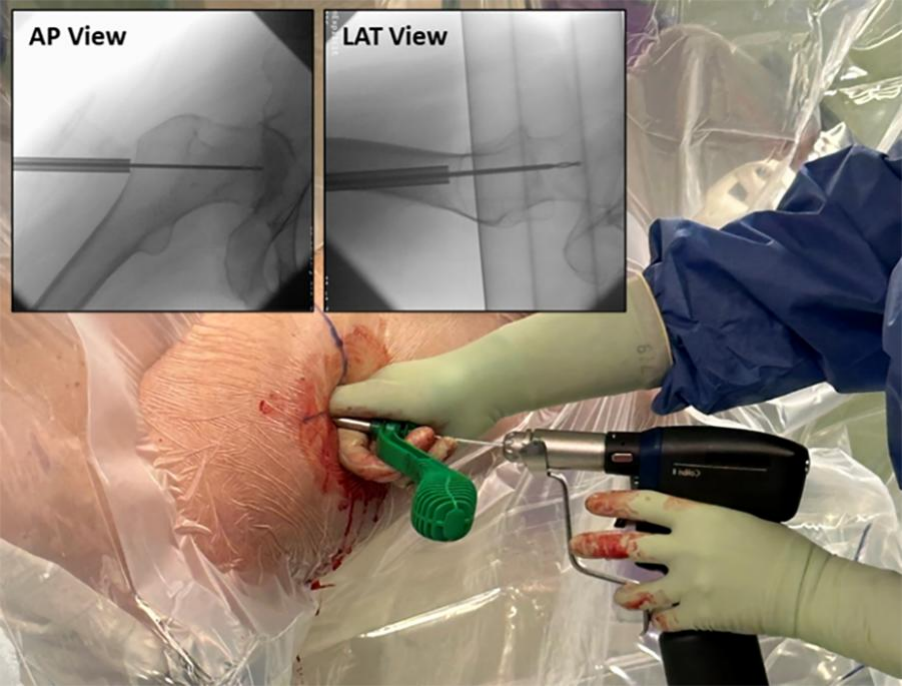
The guide pin is introduced through the obturator and tissue protector above the level of the lesser trochanter and towards the center-center position in both planes, as demonstrated by the fluoroscopic images.

**Figure 6:**
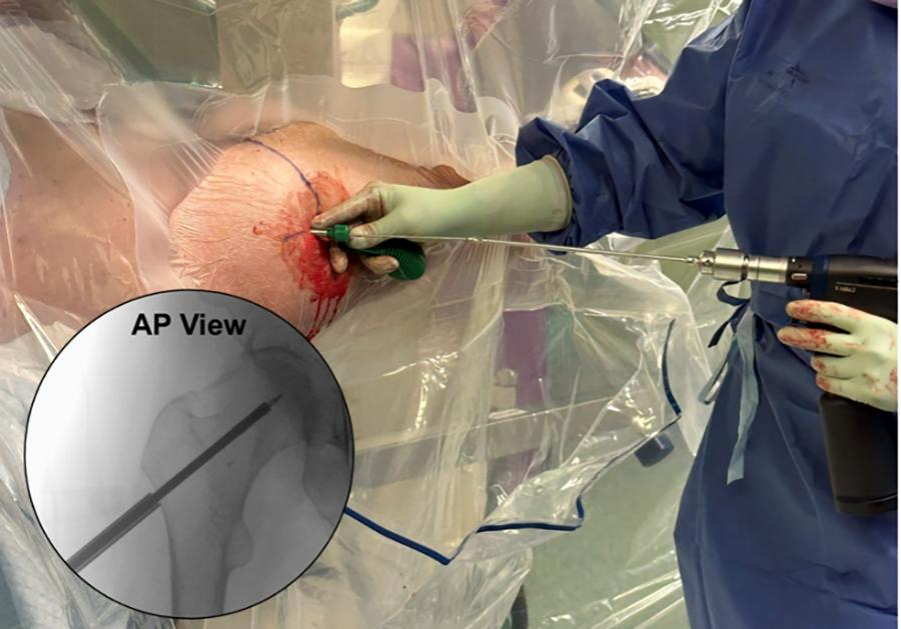
The cannulated drill is introduced over the guide pin under direct fluoroscopic visualization and advanced to the base of the femoral head.

If the soft tissue envelope is extensive, the incision can be shifted proximally by 1–3 centimeters to maintain collinearity. Final confirmation of the proper skin incision collinearity with the lateral cortical entry is accomplished using fluoroscopy during the injection of long-acting local anesthetic with epinephrine, the percentage of which is per surgeon preference. (Figure 4—Local Injection—Incision Confirmation). A 1–2 cm incision is made through the skin and Iliotibial band to the lateral cortex. The serrated tissue protector with centering obturator and 2.5 mm guide pin are introduced as a single unit to the cortex. Confirmation of a center/center trajectory is achieved by moving the serrated tissue protector and guide pin anterior and posterior on the lateral cortex. The single lateral cortical entry is made laterally along the vastus ridge at or proximal to the most superior portion of the lesser trochanter. When proper position and trajectory are confirmed on AP and lateral view, and with skin markings serving as a reference, the guide pin is inserted 1–2 cm towards the center of the femoral neck while positioning in both planes is confirmed with fluoroscopy (Figure 5—Guide pin entry). The guide pin is adjusted in both planes until the center-center position is confirmed. The guide pin is then advanced to the head–neck junction while positioning is monitored with fluoroscopy in both planes. The 5.3 mm cannulated drill is introduced to the lateral cortex and advanced over the guide pin to the base of the femoral head at the confluence of the compressive and tensile trabeculae. To avoid inadvertent advancement of the guide pin, drilling is stopped, if binding occurs (Figure 6—Drill entry).

**Figure 7:**
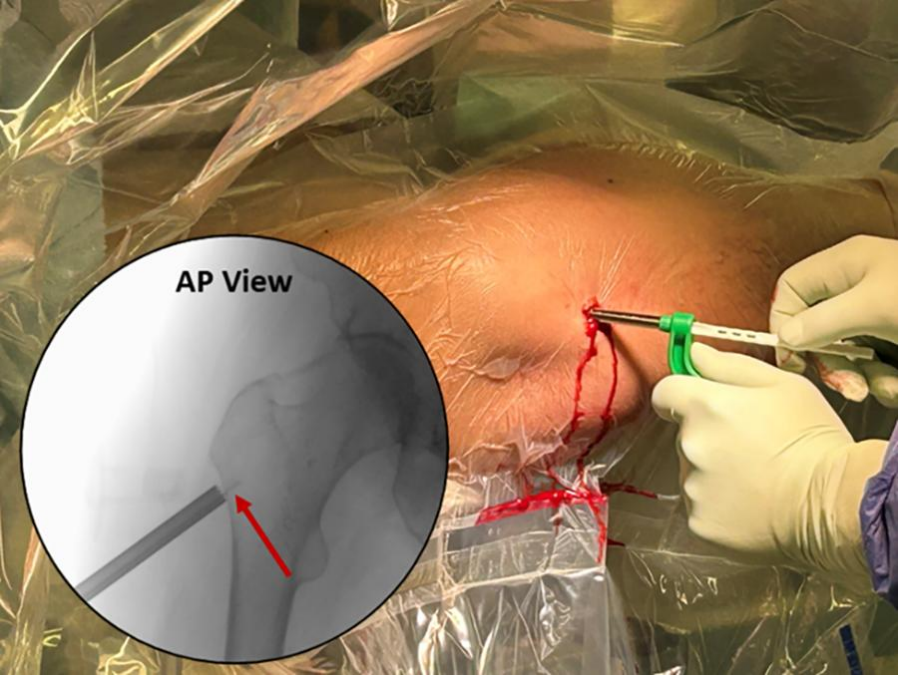
The working trough is introduced through the tissue protector, and the cutout tip is seated within the lateral cortical access portal, as demonstrated by the red arrow on the fluoroscopic image.

The tissue protector remains centered over the portal while the cannulated drill and guide pin are removed. The working trough is passed through the tissue protector and seated into the lateral portal (Figure 7—Working trough placement). If the tissue protector slips before the trough is seated in the portal, the guide pin is reinserted into the portal, and the working trough is advanced along the guide pin until it seats in the portal. Only a single lateral portal should be created; multiple entries should be avoided.

#### Debridement

Debridement helps to create a well-defined enhancement site by loosening central, non-structural elements and roughening the trabecular periphery to stimulate healing without unnecessarily thinning or violating the cortex. The curved blunt probe debrider has a flat spot on the handle that indicates tip orientation. This instrument is used to perform a three-step debridement process.

1. Loosening the central fat and nonstructural bone
2. Roughening the denser trabecular rim lightly (compressive and tensile regions) via scraping/back-scratching
3. Palpating the perimeter to create a 3-D mental map of the size and shape of the enhancement site

**Figure 8:**
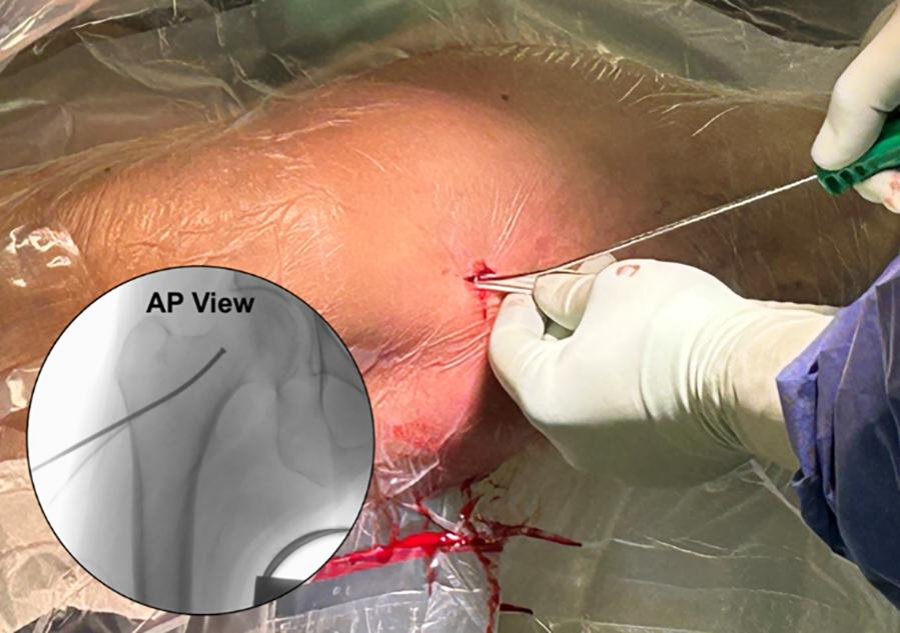
The debridement tool is introduced over the working trough and into the apex of the enhancement site. The fluoroscopic image shows the debridement tool positioned within the proximal femur.

**Figure 9:**
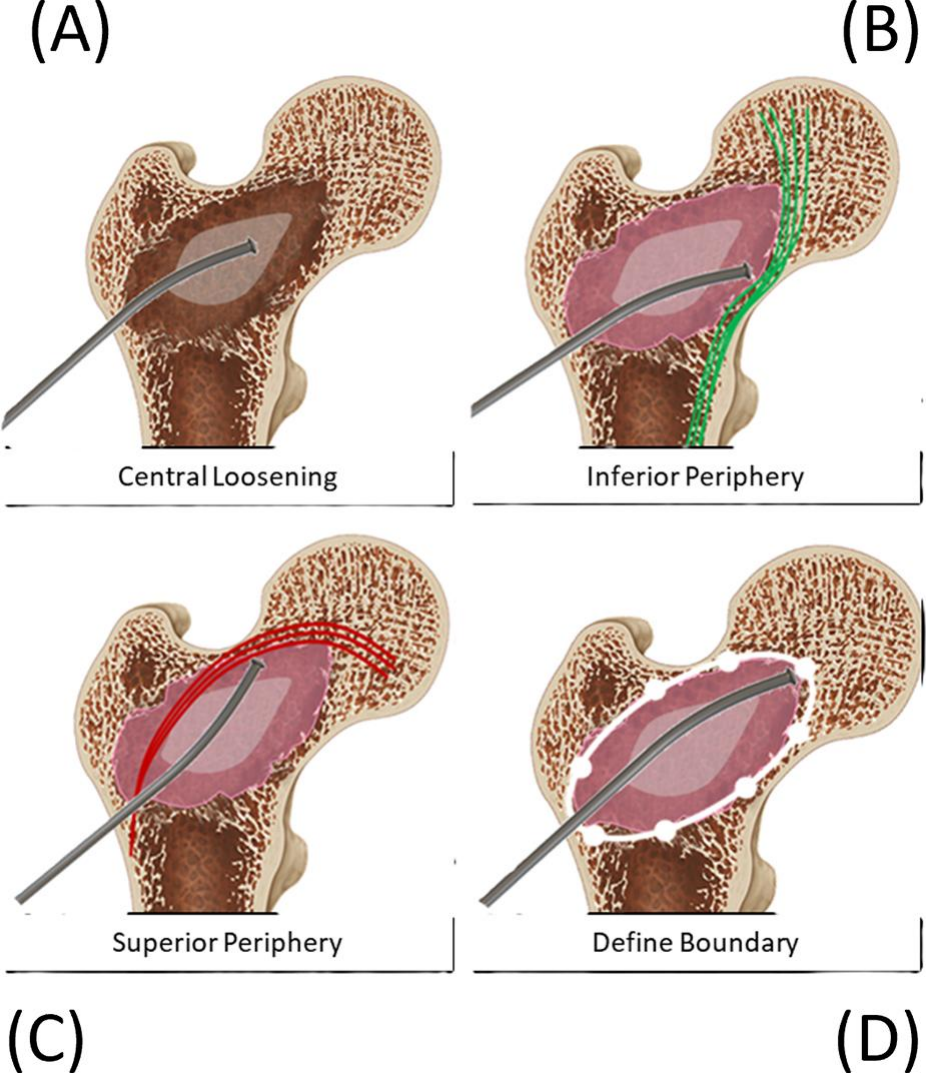
The illustration sequence demonstrates the debridement steps from central loosening **(A)** to peripheral roughening **(B,C)** and to the final probing step to define the boundaries of the enhancement site to be injected **(D)**.

The blunt probe debrider is introduced by placing the distal aspect of the device parallel with the working trough and advancing through the cortical portal. The location of the blunt probe debrider is confirmed in both planes. A gritty sensation can be perceived with the instrument when it is inside the proximal femur (Figure 8—Debridement Tool Insertion). Using fluoroscopic guidance, the surgeon works proximal to distal, loosening fatty tissue and non-structural material (Figure 9—Debridement steps). Once the central void is defined, the probe is used in a scraping or backscratching fashion to roughen the edges of the denser compressive and tensile trabeculae from medial to lateral. The cavity is then palpated with the blunt probe debrider along the edges of the void to define the perimeter to develop a three-dimensional, mental image of the enhancement site (Figure 9—Debridement steps). Material loosened during this process is not fully removed yet; removal occurs during suction/irrigation.

#### Suction/irrigation

**Figure 10:**
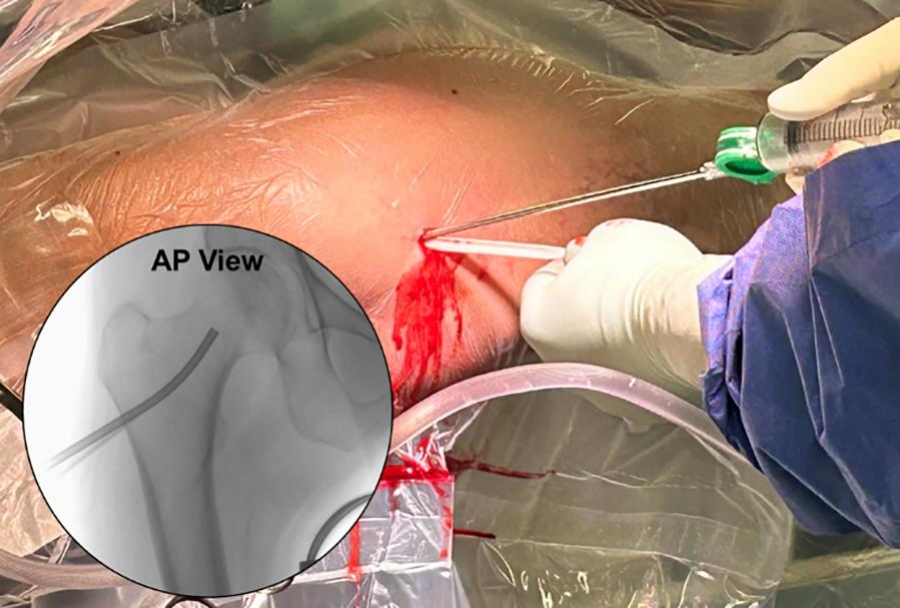
The suction/irrigation device is inserted over the working trough, and the location within the enhancement site is confirmed by fluoroscopy.

**Figure 11:**
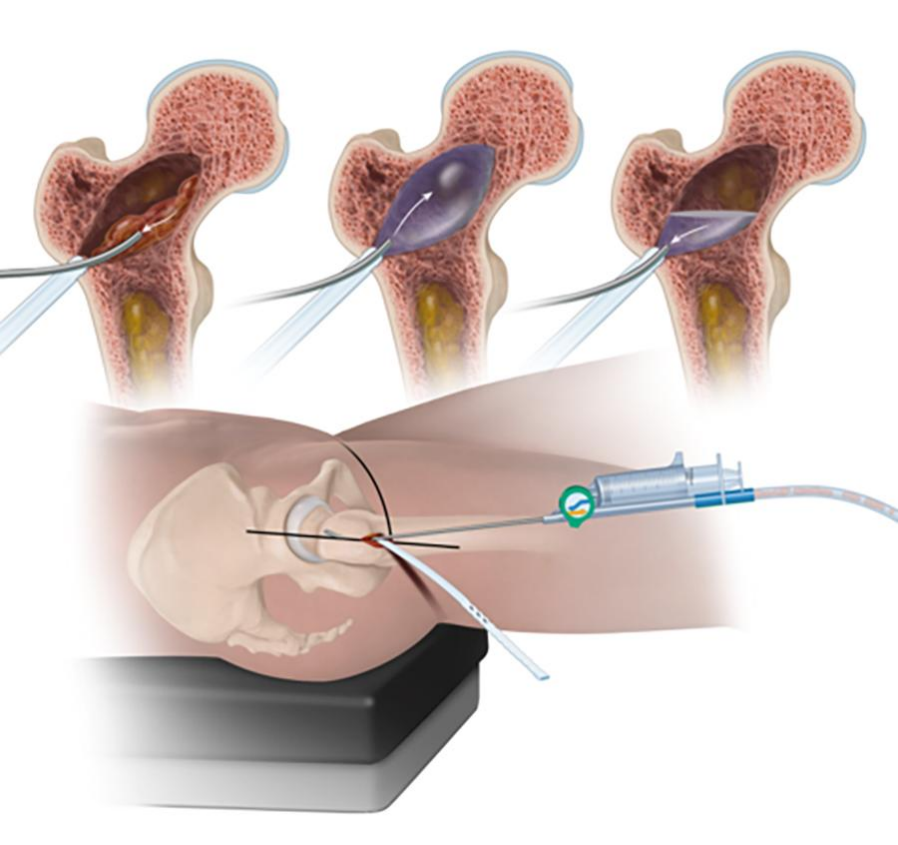
The suction/irrigation device is inserted over the working trough and confirmed within the enhancement site by fluoroscopy. During two cycles of suction and irrigation, the fat and loose nonstructural bone elements are removed.

The suction/irrigator is introduced and advanced gently along the trough to avoid coring cancellous bone (Figure 10—Insertion of suction/irrigator). If clogging occurs, the suction/irrigator is withdrawn and its tip is cleared. Initially, suction is used to remove loose bone and fatty elements, creating space and enabling a subsequent low-pressure injection of AGN1 implant material (Figure 11—Suction/Irrigation Steps). A small aliquot of 5–10 cc of sterile saline is injected into the enhancement site and suctioned immediately afterwards with care to avoid extravasation into the soft-tissue extravasation. At least two cycles of suction and irrigation are performed in this fashion, after which continuous suction of the enhancement site is maintained while the AGN1 material is mixed.

#### Mixing the AGN1 implant material

The material is mixed according to the manufacturer's instructions for use immediately before injection; the curing reaction starts upon combination of the powder components. Proper material consistency and handling properties depend on mixing the material as directed. The visual cues suggesting that mixing was successful and injection should proceed are include the paste exhibiting cohesive, non-dripping “toothpaste-like” viscosity. Note the material is radio-opaque and clearly visible on fluoroscopy. Injection should begin promptly and within the IFU handling parameters. The working time is reported to be 5–7 min by the manufacturer; temperature and time may influence viscosity. Delays between mixing and injection should be avoided to prevent premature hardening prior to injection.

#### Injection

**Figure 12:**
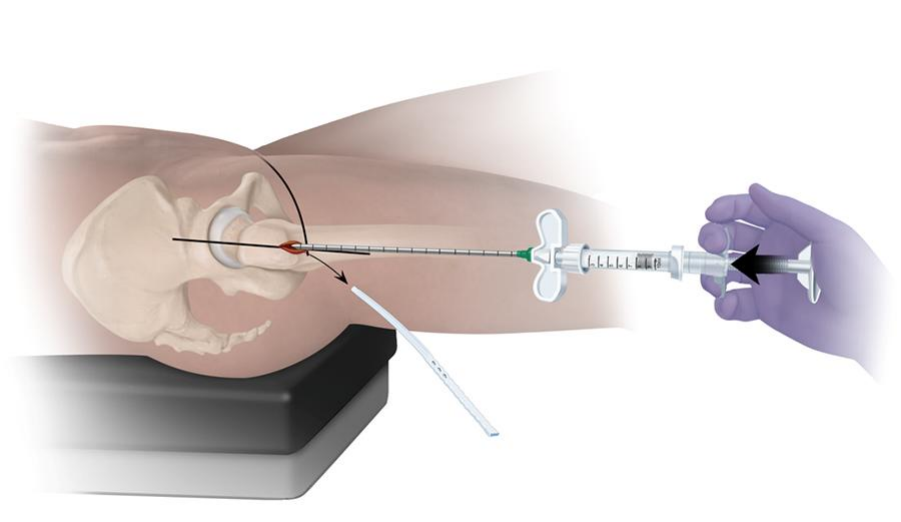
The prefilled injection cannula is inserted over the working trough, and its position within the enhancement site is confirmed by fluoroscopy. Once confirmed, the working trough is removed prior to beginning to fill the enhancement site to facilitate venting.

**Figure 13:**
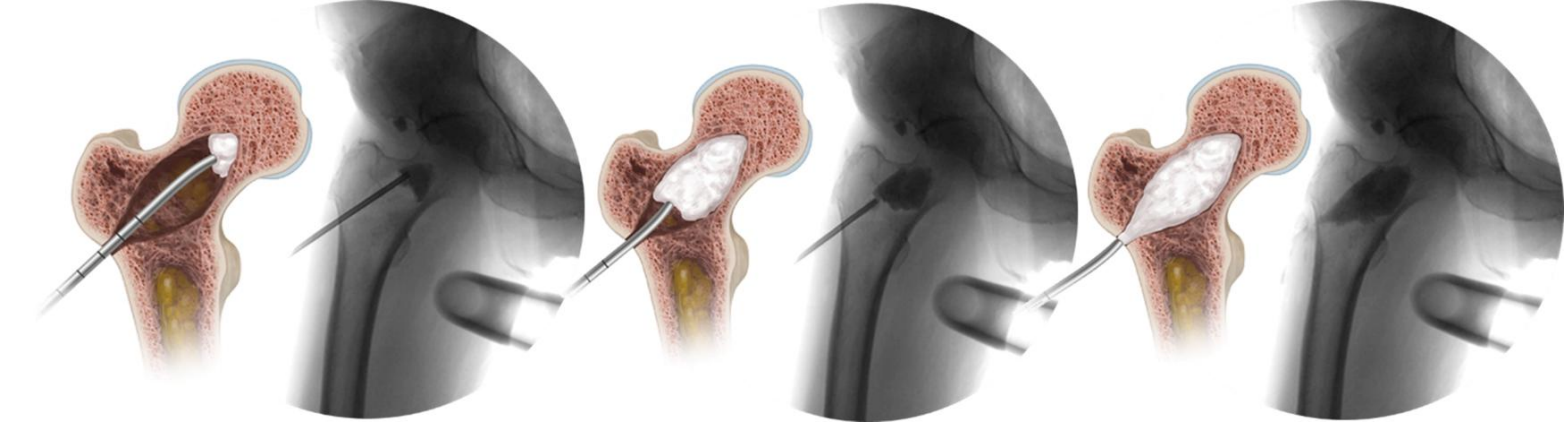
The sequence of illustrations and fluoroscopic images demonstrates the filling of the enhancement site with AGN1 from proximal to distal gradually over several minutes.

A final suction of the enhancement site is performed prior to beginning the injection. To begin, the injection cannula is advanced to the apex of the enhancement site under fluoroscopy. The working trough is removed to facilitate venting and a low-pressure injection (Figure 12—Injection Cannula Insertion). The cannula tip is retracted ∼5 mm and small aliquots (∼1–2 cc) of AGN1 are injected while the cannula tip is continuously withdrawn and repositioned so it remains on the leading edge of the bolus of material. The injection is performed slowly and under biplanar fluoroscopic monitoring to ensure complete filling while avoiding pressurization, vascular leakage, and/or extravasation (Figure 13—AGN1 filling the enhancement site). As the cannula nears the lateral portal, injection flow is reduced. At this stage, if AGN1 begins to exit the entry portal into soft tissues, the cavity is full and the injection is stopped. The goal of treatment is a complete, low-pressure fill of the site as viewed with fluoroscopy. The required volume is dependent on the size of the patient and enhancement site and should be recorded in the operative notes. The cannula is not advanced through the hardening material. If AGN1 implant material is present in soft tissues, it is gently removed with a moistened sponge and/or curette.

#### Closure

Iliotibial band/fascia are re-approximated as needed; skin is closed in standard layered fashion (e.g., subcuticular closure with adhesive strips) using sutures of surgeon's choosing. Drains have not been reported to be required.

### Special considerations for technique

Guide pin placement

**Figure 14:**
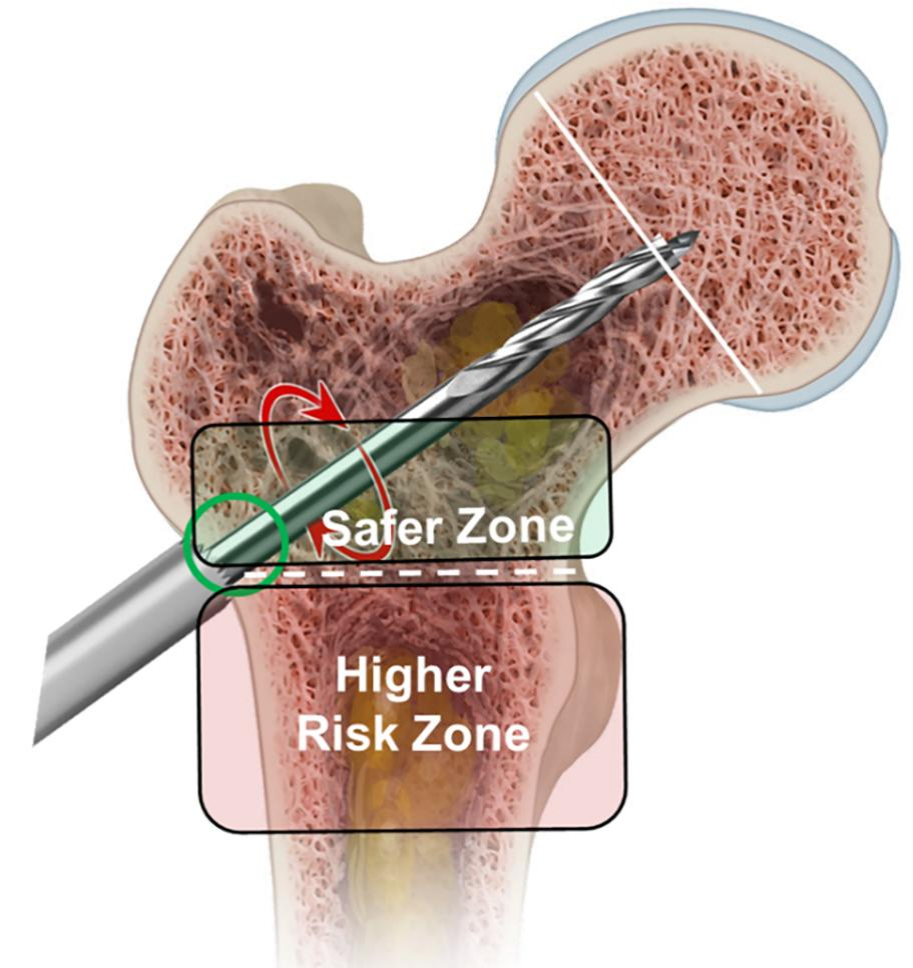
Demonstrates the guide pin and drill entering the lateral cortex above the level of the most proximal portion of the lesser trochanter in the lower risk area for stress risers.

• Entry into the lateral femoral cortex should be done only once at or proximal to the most superior level of the lesser trochanter (Figure 14—Entry zone). Entering the lateral cortex more distally may further weaken the proximal femur. If a distal entry portal is created, or if multiple entries are made in error, modification to surgical and post-surgical plans should be considered, including, but not limited to, fixation of the proximal femur and modification to weight-bearing, respectively.

Suction and irrigation

• Suction and irrigation is intended to help create space, facilitating a low-pressure injection of AGN1, reducing the risk of embolization. A suction irrigator device and suction clip are provided and used in conjunction to perform suction and irrigation. For suction, the green wheel on the suction irrigator is rotated to the forward position, the empty irrigation syringe is placed in the suction irrigator and the suction clamp is opened. The suction irrigator will not provide suction without the empty irrigation syringe being inserted. For irrigation, the green wheel is rotated to the back position, the suction clamp is closed and the irrigation syringe with 5–10 cc sterile saline is inserted. Irrigation is then performed slowly by applying gentle thumb pressure to the plunger of the irrigation syringe. Note that if the suction clamp remains open, the saline is likely to be suctioned directly from the syringe before it enters the enhancement site.

Vascular leakage

• As is the case with the use of any injectable material, there is a small risk of the material entering vasculature. The risk is minimized by performing a low-pressure, vented injection with the cannula kept on the leading edge of the bolus of material. If leakage is suspected based on fluoroscopic observation, the procedure is paused and vital signs are assessed with the anesthesiologist. Cannula repositioning and injection continues, only if the anesthesiologist and surgeon agree that the patient is stable.

Material extravasation into soft tissue

• Small amounts of material may be present in the soft tissue after the injection. A moistened sponge is used to gently remove as much material as possible. This material has been shown to resorb in the soft tissue and does not typically form bone in a heterotopic location.

Post-operative considerations

• Based on pre-clinical (40) and clinical study results (42), weight-bearing to tolerance after unilateral standalone LOEP depends on the surgeon's medical judgment and each patient's condition. Assistive devices are used as needed, and rehabilitation considerations are based on patient needs. Institutional standards are applied and patient characteristics and risk profile are considered when determining DVT/PE prophylaxis (e.g., early mobilization, mechanical prophylaxis, and chemoprophylaxis such as LMWH or a DOAC per guideline and renal function). If a patient is frail, if multiple entry holes were made, the entry location was made too distal, and/or the patient has pain with weight-bearing in the operated hip after surgery, precautionary measures and modified weight-bearing are considered until the absence of a fracture is confirmed.

Performing LOEP concomitant with acute hip fracture surgery

**Figure 15:**
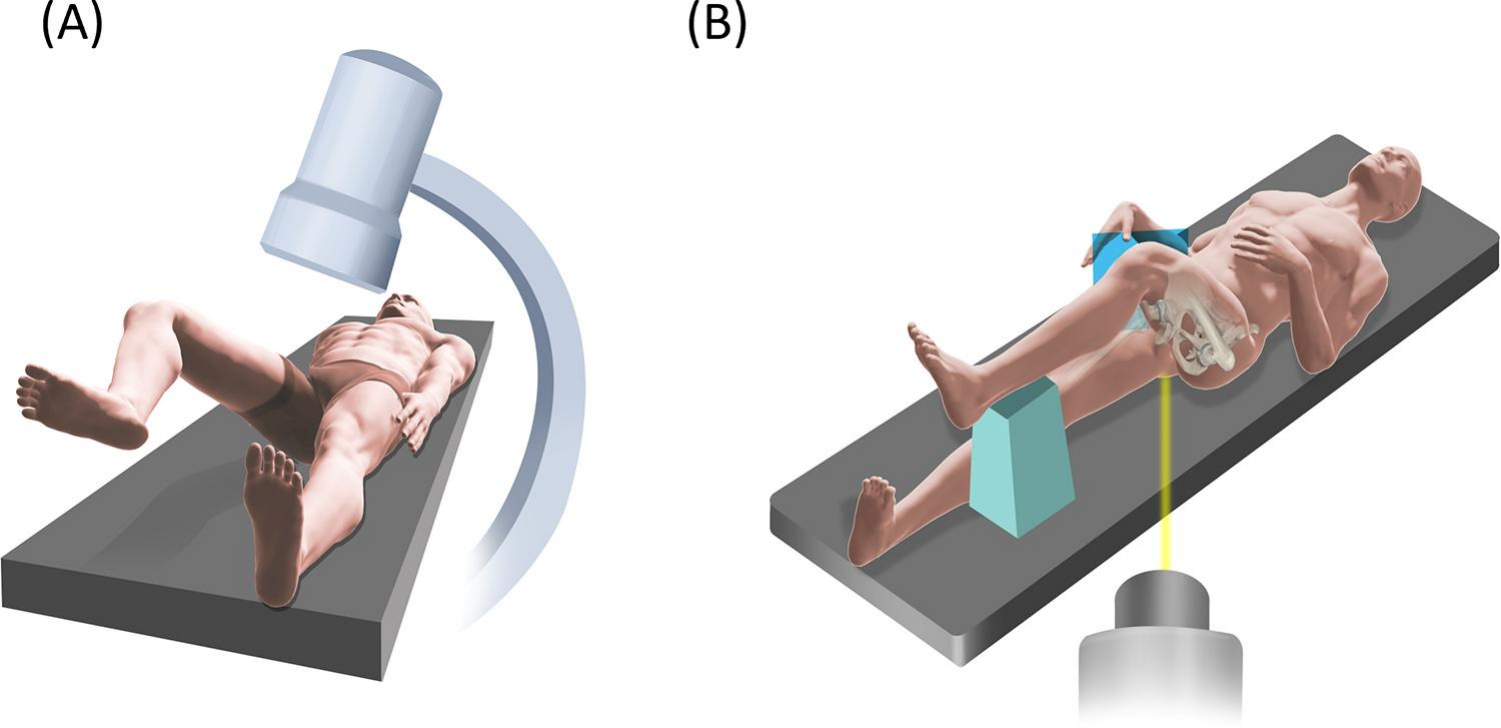
Illustration of positioning techniques to obtain the lateral fluoroscopy views for patients undergoing contralateral hip replacement for fracture repair prior to LOEP. Lateral views can be achieved via the frog leg position **(A)** or a cross-table acquisition with the contralateral hip flexed at 90° **(B)**.

• Patients presenting with an acute hip fragility fracture are likely to be in a more fragile state than those treated in an elective setting. As such, the surgeon relies upon product labeling and his/her judgment to determine the appropriateness of contralateral LOEP treatment of the non-fractured proximal femur. If LOEP is performed in this setting, as during the ongoing RESTORE study, several factors are considered.
• At the end of hip fracture surgery, LOEP should only proceed if the patient is hemodynamically stable and the team, including anesthesiologist, are comfortable with extended anesthesia and operative time.
• LOEP treatment contralateral to hip replacement to treat a femoral neck fracture:
  ○ If the patient is undergoing a hip replacement in the supine position, both legs are draped simultaneously, and no repositioning is performed. Here, the alteration of the technique described above involves obtaining the lateral view from the frog leg position or cross-table acquisition. (Figure 15—Patient Positioning Following Hip Replacement in Supine Position)
  ○ If the patient is undergoing hip replacement via a posterolateral approach, he/she is repositioned on the fracture table. Care is taken when positioning the operated hip to avoid hip dislocation. The procedure then commences as described above.
• LOEP treatment contralateral to osteosynthesis:
  ○ If using a fracture table, significant time is saved when the fracture table is adjusted immediately after the last implant/screw have been placed and the wound is closed. Otherwise, the procedure remains as described above.

Post-LOEP surgical procedures

• Clinical experience and bench top testing have shown that drilling or sawing with standard surgical tools is feasible peri-operatively or even later, once some or all of the AGN1 material has been resorbed and replaced with bone.

## Results

### Completed clinical studies

The following section summarizes two completed and published clinical studies evaluating AGN1 LOEP in postmenopausal women at risk of fragility fracture. The primary focus for both studies was assessing safety and feasibility. Secondary analyses were performed to assess AGN1 resorption, new bone formation, and Bone Mineral Density (BMD).

1. First-in-Human Clinical Feasibility and Safety Study (Copley)—(**NCT06799143**)

This prospective, non-blinded, single-center feasibility study was conducted at Copley Hospital (USA) and enrolled 12 Caucasian, post-menopausal women (mean age = 71.7, range 56–89) with osteoporosis, as confirmed by baseline femoral neck DXA T-scores ≤–2.5 (Hologic Delphi C). The mean baseline femoral neck T-score of participants was −2.9 ± 0.4. Each participant's left hip was treated with AGN1 LOEP, while the right hip served as an untreated control. DXA scans were performed at baseline, 1, 6, 12, 18 and 24 weeks, 12, 18, 24 months and at the extension timepoint at an average of 6 years post treatment. Radiographs were performed at baseline, 6, 12, 24 weeks and at the extension timepoint. Quantitative computed tomography (QCT) was performed at baseline, 12 and 24 weeks and at the extension timepoint. Average implant volume was 19 ± 2 cc (range 15–22 cc). The percentage resorption of AGN1 was estimated via serial QCT by comparing the initial intense radiopaque implant region of interest (ROI) with subsequent appearance in the same ROI of lower density. At 24 weeks, only 17 ± 14% of the area of intense radiopacity remained. Newly formed bone was observed replacing the material in all participants. Mean femoral neck BMD of treated hips was 68 ± 22% higher than that of untreated control hips at 12 months, 59 ± 24% higher at 24 months, and 58 ± 27% higher at the extension timepoint. Finite Element Analysis confirmed a persistent increase in femoral neck strength in the treated hips compared with controls. There were no procedure or device causally-related serious adverse events (SAEs). Of the ten adverse events reported among five participants, three were at least possibly related to the procedure (i.e., small area of wound breakdown, irritation injection, and post-operative nausea). Seven fragility fractures occurred in 4 subjects, including three hip fractures. Two hip fractures occurred in untreated, control hips (27 and 44 months after treatment) and one in a treated hip (40 months after treatment). No thromboembolic or cardiovascular events were reported during follow-up (43).

2. Proof of Concept Study for LOEP Concomitant with Hip Fragility Fracture Surgery (STRONG)—(**NCT03268356**)

This prospective, non-blinded, single-center study at the University of Hong Kong evaluated the feasibility of contralateral LOEP in women undergoing surgery for an index hip fragility fracture. Thirteen post-menopausal women >65 years of age (median 83 years) provided written consent and underwent standard fixation (*N* = 8) or arthroplasty (*N* = 5) of the fractured hip followed by contralateral AGN1 LOEP treatment in the same operative session. Since participants presented with a hip fracture, baseline DXA scans were not feasible. Only two of the participants had been diagnosed with osteoporosis prior to the study. The mean implant volume was 14.7 ± 2.7 mL. Treatment added an average of 24.9 min to total surgical time, including repositioning. Participants followed the hospital's standard hip fracture rehabilitation pathway without modification for LOEP. Radiographs demonstrated progressive resorption of AGN1 with replacement by new bone within 12 months in all participants. The total hip mean BMD T-score results were −1.32 (SD: 1.01) and −1.48 (SD: 1.22) at 12 and 24 months, respectively. Throughout the 24-month follow-up, no contralateral fractures occurred in treated hips. There was one mortality due to ischemic heart disease adjudicated to be unrelated to the study material and possibly related to the hip fracture repair and/or LOEP procedure. An autopsy was performed and no thrombo-emboli or foreign bodies were found in the pulmonary arteries or veins, femoral veins, iliac veins, inferior vena cava or lungs. Two other SAEs of moderate severity were listed as unlikely related to the procedure (i.e., post-operative pneumonia and subdural hematoma) (42).

#### Summary

These studies support the safety and technical feasibility of AGN1 LOEP as a standalone, elective treatment and when performed concomitantly with surgery to treat a fragility hip fracture in the same operative session. Consistent results were observed including complete material resorption and replacement with new bone as well as increased bone density and strength.

## Discussion

In the early 1900s, Sir Reginald Watson-Jones, an academic orthopedic surgeon, was credited with saying that “you are born through the brim of the pelvis, and you leave through the hip”. Unfortunately, this statement remains as true today as it was then. Despite major advances in surgical technique, fixation devices, perioperative care, and pharmacologic treatments, fragility fractures—particularly those of the hip—remain a persistent global challenge (17). The annual incidence of hip fractures is estimated to have exceeded 14 million in 2019, with further increases projected worldwide (6, 15, 16). For healthcare systems, the economic burden is substantial, while for patients and their families, hip fractures result in loss of independence, diminished quality of life and excess mortality (6). One-year mortality rates, following surgical stabilization of hip fracture, have been reported as 37% in men and 28% in women (47). Furthermore, up to 10% of patients experience a contralateral hip fracture within one year of the index event, and these second fractures are associated with even poorer outcomes (48, 49).

There is broad consensus across national and international guidelines that osteoporosis pharmacotherapy after an index fracture is essential (50). Unfortunately, the treatment gap remains high. Even in patients with a hip fracture, treatment is initiated within a year only 20% of the time (51, 52). Initiating pharmacologic treatment for osteoporosis remains difficult for several reasons. Patients and physicians often fail to recognize osteoporosis as it is a silent disease until a fracture occurs. Contributing factors include diagnostic delays, limited post-fracture follow-up programs, patient reluctance and concerns about the side effects of medication. Even when treatment begins, real-world adherence is low (<50% after 12 months), substantially limiting effectiveness (53, 54). Pharmacotherapies such as bisphosphonates, denosumab, and anabolic agents can increase femoral-neck BMD by 2%–6% and some reduce hip fracture risk by up to approximately 50% in placebo-controlled trials, but their benefit becomes measurable at the hip only after 12–18 months of therapy (24, 27, 30, 55–58). Therefore, while pharmacotherapy remains essential, it cannot close the post-fracture treatment gap by itself. This may explain why LOEP appears in the European guidance for the diagnosis and management of osteoporosis in postmenopausal women as developed by the scientific advisory board of the European Society for Clinical and Economic Aspects of Osteoporosis (ESCEO) as well as the committee of scientific advisors and national societies of the International Osteoporosis Foundation (IOF) (50). Orthopedic surgeons, positioned at the interface of acute fracture care and prevention, may therefore play a crucial role in developing targeted, preventative surgical strategies to reduce the burden of future fractures.

### Proximal femur augmentation and historical limitations

Augmentation of the proximal femur has been investigated for decades using materials such as poly-methyl methacrylate (PMMA), calcium phosphate, micro-glass (Cortoss®) and polyetheretherketone (PEEK Y-strut®) (40, 59–62). These approaches demonstrated mechanical reinforcement in cadaveric models, but most materials are inert, non-resorbable and may produce thermal necrosis or modulus mismatch with surrounding bone (63–68). Heini et al. were among the first to explore standalone PMMA augmentation of the femoral neck and intertrochanteric regions of cadaveric femurs. Heini injected until leakage was observed or a volume of 41 mL was injected, whichever occurred first. The mean volume was 36 mL (range = 28–41 mL). Heini demonstrated improved mechanical stability but also recorded a 20–30 °C rise in local bone surface temperature, risking thermal damage to the periosteal blood supply and potential avascular necrosis *in vivo* (69). The study involved a limited sample size, and while it established feasibility, its findings underscore the limitations of PMMA as a non-resorbable, exothermic material. Subsequent biomechanical and computational analyses have confirmed that augmentation materials can increase the load-to-failure of osteoporotic femurs, yet none of these techniques have been widely adopted clinically (59, 67–70). Reasons are likely related to invasiveness of cortical perforation, the risk of fracture propagation and the incompatibility of many materials with subsequent arthroplasty. As Varga et al. emphasized, successful femoral augmentation should fulfill the following criteria: minimal invasiveness, immediate mechanical strengthening, preservation of future surgical options, and clinical and financial feasibility (71).

Although LOEP involves a cortical entry, it remains minimally invasive in the surgical sense, as the incision and bone access are limited to a single, small, fluoroscopically guided portal in the lateral cortex at or proximal to the most superior level of the lesser trochanter, with no intramedullary or joint violation. The risk of iatrogenic fracture is minimized through low-pressure injection, single cortical entry in proper location, and fluoroscopic monitoring throughout the procedure.

### Immediate and long-term biomechanical effects

Stroncek et al. evaluated the biomechanical impact of LOEP using 45 matched pairs of cadaver femurs treated with AGN1 on one side compared with the contralateral untreated control (40). All femurs underwent DXA assessment and were grouped into osteoporotic, osteopenic and normal cohorts. Using a validated sideways fall model for human cadaver femurs involving compressive testing to failure, the cohort of 25 matched pairs of osteoporotic femurs showed a 23% increase in peak load to failure and 30% increase in energy to failure 24 h after treatment—the time required for complete curing of the material. Modulus of elasticity did not change statistically, which is expected to help preserve physiologic load distribution. These results reflect short-term enhancement of biomechanical properties of treated femurs (40).

### Resorption, remodeling and clinical correlation

The defining feature of LOEP is the resorption and replacement of the AGN1 material with new bone. In both preclinical and clinical studies, the material was shown to be substantially resorbed and replaced by bone within 12 months (42–45). This biological remodeling differentiates this treatment from prior augmentation techniques that relied upon inert materials. The ability of AGN1 to be remodeled and integrate with host bone supports the prospects for its long-terms safety and compatibility with potential future hip procedures.

### Current clinical evidence and future directions

All completed clinical studies have demonstrated that LOEP is technically feasible, safe and biologically compatible, yielding sustained increases in local bone density and strength as measured by BMD and FEA (42, 43). Although these preliminary data suggest that a reduction in fracture risk is possible, direct clinical proof from randomized, controlled studies is pending. The ongoing RESTORE randomized, controlled trial (NCT04796350) aims to fill this gap in evidence by determining whether contralateral LOEP treatment in patients with an index hip fracture can reduce the cumulative incidence of secondary hip fractures during five-years follow-up. RESTORE has been initiated in over 60 sites across Europe, Japan, and Canada, and has enrolled more than 400 participants to date.

Economic analyses should be interpreted cautiously. While a modeling study has proposed that only a 2.8% reduction in hip fractures would render prophylactic augmentation cost-effective (72), such data remain hypothetical until confirmed by randomized outcomes. Future cost-benefit conclusions will depend on evidence generated by RESTORE and/or similar large-scale studies.

## Conclusion

LOEP offers a promising, biologically compatible approach to treating local bone loss, thereby helping to address hip fragility. The treatment addresses key limitations of prior augmentation techniques and may help to address persistent unmet needs in fracture prevention. While early clinical results are encouraging, data from a large, long-term, randomized study are necessary to confirm its impact on fracture incidence, safety, cost-effectiveness and feasibility for incorporation into standard-of-care treatment protocols.

## Data Availability

The original contributions presented in the study are included in the article/Supplementary Material, further inquiries can be directed to the corresponding author.

## Author's note

Local Osteo-Enhancement Procedure delivers AGN1 implant material which is resorbed and replaced by bone, significantly increasing the strength of the proximal femur.

## References

[1] Verhulp E van Rietbergen B Huiskes R. Load distribution in the healthy and osteoporotic human proximal femur during a fall to the side. Bone. (2008) 42:30–5. 10.1016/j.bone.2007.08.039 17977813

[2] Kanis JA, WHO Study Group. Assessment of fracture risk and its application to screening for postmenopausal osteoporosis: synopsis of a WHO report. Osteoporos Int. (1994) 4:368–81. 10.1007/BF01622200 7696835

[3] Xiao PL Cui AY Hsu CJ Peng R Jiang N Xu XH Global, regional prevalence, and risk factors of osteoporosis according to the World Health Organization diagnostic criteria: a systematic review and meta-analysis. Osteoporos Int. (2022) 33:2137–53. 10.1007/s00198-022-06454-3 35687123

[4] Sözen T Özışık L Çalık Başaran N. An overview and management of osteoporosis. Eur J Rheumatol. (2017) 4:46–56. 10.5152/eurjrheum.2016.048 28293453 PMC5335887

[5] Marshall D Johnell O Wedel H. Meta-analysis of how well measures of bone mineral density predict occurrence of osteoporotic fractures. Br Med J. (1996) 312:1254–9. 10.1136/bmj.312.7041.1254 8634613 PMC2351094

[6] Svedbom A Hernlund E Ivergård M Compston J Cooper C Stenmark J Osteoporosis in the European union: a compendium of country-specific reports. Arch Osteoporos. (2013) 8:137. 10.1007/s11657-013-0137-0 24113838 PMC3880492

[7] World Health Organization. Fragility fractures (2024). Available online at: https://www.who.int/news-room/fact-sheets/detail/fragility-fractures (Accessed 22 October 2025).

[8] Abrahamsen B van Staa T Ariely R Olson M Cooper C. Excess mortality following hip fracture: a systematic epidemiological review. Osteoporos Int. (2009) 20:1633–50. 10.1007/s00198-009-0920-3 19421703

[9] Borgstrom F Zethraeus N Johnell O Lidgren L Ponzer S Svensson O Costs and quality of life associated with osteoporosis-related fractures in Sweden. Osteoporos Int. (2006) 17:637–50. 10.1007/s00198-005-0015-8 16283064

[10] Burge R Dawson-Hughes B Solomon DH Wong JB King A Tosteson A. Incidence and economic burden of osteoporosis-related fractures in the United States, 2005–2025. J Bone Miner Res. (2007) 22:465–75. 10.1359/jbmr.061113 17144789

[11] Office of the Surgeon General (US). Bone Health and Osteoporosis: A Report of the Surgeon General. MD: Rockville (2004). 20945569

[12] Boutroy S Khosla S Sornay-Rendu E Zanchetta MB McMahon DJ Zhang CA Microarchitecture and peripheral BMD are impaired in postmenopausal white women with fracture independently of total hip T-score: an international multicenter study. J Bone Miner Res. (2016) 31:1158–66. 10.1002/jbmr.2796 26818785 PMC4891284

[13] Khosla S Cauley JA Compston J Kiel DP Rosen C Saag KG Addressing the crisis in the treatment of osteoporosis: a path forward. J Bone Miner Res. (2017) 32:424–30. 10.1002/jbmr.3074 28099754

[14] Wa P. Consensus development conference: diagnosis, prophylaxis, and treatment of osteoporosis. Am J Med. (1993) 94:646–50. 10.1016/0002-9343(93)90218-e 8506892

[15] Dong Y Zhang Y Song K Kang H Ye D Li F. What was the epidemiology and global burden of disease of hip fractures from 1990 to 2019? Results from and additional analysis of the global burden of disease study 2019. Clin Orthop Relat Res. (2023) 481:1209–20. 10.1097/CORR.0000000000002465 36374576 PMC10194687

[16] Gullberg B Johnell O Kanis JA. World-wide projections for hip fracture. Osteoporos Int. (1997) 7:407–13. 10.1007/pl00004148 9425497

[17] Khosla S Cauley JA Compston J Kiel DP Rosen C Saag KG Addressing the crisis in the treatment of osteoporosis: a path forward. J Bone Miner Res. (2017) 32(3):424–30. 10.1002/jbmr.3074 28099754

[18] Ferrari S Reginster JY Brandi ML Kanis JA Devogelaer JP Kaufman JM Unmet needs and current and future approaches for osteoporotic patients at high risk of hip fracture. Arch Osteoporos. (2016) 11:37. 10.1007/s11657-016-0292-1 27800591 PMC5306171

[19] Khosla S Shane E. A crisis in the treatment of osteoporosis. J Bone Miner Res. (2016) 31:1485–7. 10.1002/jbmr.2888 27335158

[20] Abrahamsen B Brask-Lindemann D Rubin KH Schwarz P. A review of lifestyle, smoking and other modifiable risk factors for osteoporotic fractures. Bonekey Rep. (2014) 3:574. 10.1038/bonekey.2014.69 25228987 PMC4162465

[21] Body JJ Bergmann P Boonen S Boutsen Y Bruyere O Devogelaer JP Non-pharmacological management of osteoporosis: a consensus of the Belgian Bone Club. Osteoporos Int. (2011) 22:2769–88. 10.1007/s00198-011-1545-x 21360219 PMC3186889

[22] Cosman F de Beur SJ LeBoff MS Lewiecki EM Tanner B Randall S Clinician’s guide to prevention and treatment of osteoporosis. Osteoporos Int. (2014) 25:2359–81. 10.1007/s00198-014-2794-2 25182228 PMC4176573

[23] Li H Xiao Z Quarles LD Li W. Osteoporosis: mechanism, molecular target and current status on drug development. Curr Med Chem. (2021) 28:1489–507. 10.2174/0929867327666200330142432 32223730 PMC7665836

[24] Lyles KW Colón-Emeric CS Magaziner JS Adachi JD Pieper CF Mautalen C Zoledronic acid and clinical fractures and mortality after hip fracture. N Engl J Med. (2007) 357:1799–809. 10.1056/NEJMoa074941 17878149 PMC2324066

[25] McClung MR Geusens P Miller PD Zippel H Bensen WG Roux C Effect of risedronate on the risk of hip fracture in elderly women. N Engl J Med. (2001) 344:333–40. 10.1056/NEJM200102013440503 11172164

[26] Eastell R Black DM Boonen S Adami S Felsenberg D Lippuner K Effect of once-yearly zoledronic acid five milligrams on fracture risk and change in femoral neck bone mineral density. J Clin Endocrinol Metab. (2009) 94:3215–25. 10.1210/jc.2008-2765 19567517 PMC5101059

[27] Neer RM Arnaud CD Zanchetta JR Prince R Gaich GA Reginster JY Effect of parathyroid hormone (1–34) on fractures and bone mineral density in postmenopausal women with osteoporosis. N Engl J Med. (2001) 344:1434–41. 10.1056/NEJM200105103441904 11346808

[28] Black DM Delmas PD Eastell R Reid IR Boonen S Cauley JA Once-yearly zoledronic acid for treatment of postmenopausal osteoporosis. N Engl J Med. (2007) 356:1809–22. 10.1056/NEJMoa067312 17476007

[29] Reginster JY Seeman E De Vernejoul MC Adami C Compston J Phenekos C Strontium ranelate reduces the risk of nonvertebral fractures in postmenopausal women with osteoporosis: treatment of peripheral osteoporosis (TROPOS) study. J Clin Endocrinol Metab. (2005) 90:2816–22. 10.1210/jc.2004-1774 15728210

[30] Chesnut CH Ettinger MP Miller PD Baylink DJ Emkey R Harris ST Ibandronate produces significant, similar antifracture efficacy in North American and European women: new clinical findings from BONE. Curr Med Res Opin. (2005) 21:391–401. 10.1185/030079905X30752 15811208

[31] Black DM Cummings SR Karpf DB Cauley JA Thompson DE Nevitt MC Randomised trial of effect of alendronate on risk of fracture in women with existing vertebral fractures. Lancet. (1996) 348:1535–41. 10.1016/s0140-6736(96)07088-2 8950879

[32] Ettinger B Black DM Mitlak BH Knickerbocker RK Nickelsen T Genant HK Reduction of vertebral fracture risk in postmenopausal women with osteoporosis treated with raloxifene: results from a 3-year randomized clinical trial. Multiple outcomes of raloxifene evaluation (MORE) investigators. JAMA. (1999) 282:637–45. 10.1001/jama.282.7.637 10517716

[33] Cauley JA Robbins J Chen Z Cummings SR Jackson RD LaCroix AZ Effects of estrogen plus progestin on risk of fracture and bone mineral density: the women’s health initiative randomized trial. JAMA. (2003) 290:1729–38. 10.1001/jama.290.13.1729 14519707

[34] Warriner AH Curtis JR. Adherence to osteoporosis treatments: room for improvement. Curr Opin Rheumatol. (2009) 21:356–62. 10.1097/BOR.0b013e32832c6aa4 19412103 PMC2913429

[35] Cramer JA Amonkar MM Hebborn A Altman R. Compliance and persistence with bisphosphonate dosing regimens among women with postmenopausal osteoporosis. Curr Med Res Opin. (2005) 21:1453–60. 10.1185/030079905X61875 16197664

[36] Fatoye F Smith P Gebrye T Yeowell G. Real-world persistence and adherence with oral bisphosphonates for osteoporosis: a systematic review. BMJ Open. (2019) 9:e027049. 10.1136/bmjopen-2018-027049 30987990 PMC6500256

[37] Parker MJ Gillespie WJ Gillespie LD. Effectiveness of hip protectors for preventing hip fractures in elderly people: systematic review. Br Med J. (2006) 332:571–4. 10.1136/bmj.38753.375324.7C 16513687 PMC1397761

[38] Piccirilli E Cariati I Primavera M Triolo R Gasbarra E Tarantino U. Augmentation in fragility fractures, bone of contention: a systematic review. BMC Musculoskelet Disord. (2022) 23:1046. 10.1186/s12891-022-06022-0 36457070 PMC9717408

[39] Yoo J Ma X Lee J Hwang J. Research update on stress riser fractures. Indian J Orthop. (2021) 55:560–70. 10.1007/s43465-020-00291-4 33995860 PMC8081793

[40] Stroncek JD Shaul JL Favell D Hill RS Huber BM Howe JG *In vitro* injection of osteoporotic cadaveric femurs with a triphasic calcium-based implant confers immediate biomechanical integrity. J Orthop Res. (2019) 37:908–15. 10.1002/jor.24239 30793358 PMC6593990

[41] Chin M Hill R Huber B Howe J Engelke K. AGN1 Local osteo-enhancement procedure increases proximal femur volumetric bone mineral density of women with post-menopausal osteoporosis as assessed by quantitative computed tomography analysis. JBMR Plus. (2025) 9:ziaf036. 10.1093/jbmrpl/ziaf036 40297188 PMC12035697

[42] Fang C Fang E Yee DKH Lau TW Cheung J Leung F. AGN1 Local osteo-enhancement procedure for treatment of contralateral proximal femur bone loss in post-menopausal osteoporosis during acute hip fracture repair with two-year follow-up. Int J Bone Frag. (2024) 4:112–8. 10.57582/IJBF.240403.112

[43] Howe JG Hill RS Stroncek JD Shaul JL Favell D Cheng RR Treatment of bone loss in proximal femurs of postmenopausal osteoporotic women with AGN1 local osteo-enhancement procedure (LOEP) increases hip bone mineral density and hip strength: a long-term prospective cohort study. Osteoporos Int. (2020) 31:921–9. 10.1007/s00198-019-05230-0 31802158 PMC7170985

[44] Shaul J Hill R Bruder S Tilton A Howe J. Triphasic calcium-based implant material resorbs and is replaced with bone in ovariectomized rats with or without bisphosphonate treatment. J Orthop Res. (2022) 40:2271–80. 10.1002/jor.25255 34935182

[45] Shaul JL Hill RS Bouxsein ML Burr DB Tilton AK Howe JG. AGN1 Implant material to treat bone loss: resorbable implant forms normal bone with and without alendronate in a canine critical size humeral defect model. Bone. (2022) 154:116246. 10.1016/j.bone.2021.116246 34744020

[46] Instructions for Use. OSSURE^TM^ local osteo-enhancement procedure (LOEP) kit. Available online at: https://www.agnovos.com/assets/pdf/AGN1-PRT-0087-rev-9-all-languages.pdf (Accessed October 30, 2025).

[47] Schnell S Friedman SM Mendelson DA Bingham KW Kates Stephen L. The 1-year mortality of patients treated in a hip fracture program for elders. Geriatr Orthop Surg Rehabil. (2010) 1:6–14. 10.1177/2151458510378105 23569656 PMC3597289

[48] Ryg J Rejnmark L Overgaard S Brixen K Vestergaard P. Hip fracture patients at risk of second hip fracture: a nationwide population-based cohort study of 169,145 cases during 1977–2001. J Bone Miner Res. (2009) 24:1299–307. 10.1359/jbmr.090207 19257816

[49] Holt G Smith R Duncan K Hutchison JD Gregori A Reid D. Outcome after sequential hip fracture in the elderly. J Bone Joint Surg Am. (2012) 94:1801–8. 10.2106/JBJS.J.01539 23032591

[50] Kanis JA Cooper C Rizzoli R Reginster JY, Scientific Advisory Board of the European Society for Clinical, Economic Aspects of Osteoporosis and Osteoarthritis (ESCEO), et al. Executive summary of the European guidance for the diagnosis and management of osteoporosis in postmenopausal women. Calcif Tissue Int. (2019) 104:235–8. 10.1007/s00223-018-00512-x 30796490 PMC6422308

[51] Kanis JA Svedbom A Harvey N McCloskey EV. The osteoporosis treatment gap. J Bone Miner Res. (2014) 29:1926–8. 10.1002/jbmr.2301 24956507

[52] Galli S Weiss D Beck A Scerpella T. Osteoporosis care gap after hip fracture—worse with low healthcare access and quality. J Clin Densitom. (2022) 25:424–31. 10.1016/j.jocd.2021.09.002 34696980

[53] Bouxsein ML Eastell R Lui LY Wu LA de Papp AE Grauer A Change in bone density and reduction in fracture risk: a meta-regression of published trials. J Bone Miner Res. (2019) 34:632–42. 10.1002/jbmr.3641 30674078

[54] Solomon DH Avorn J Katz JN Finkelstein JS Arnold M Polinski JM Compliance with osteoporosis medications. Arch Intern Med. (2005) 165:2414–9. 10.1001/archinte.165.20.2414 16287772

[55] Cummings SR Martin J McClung MR Siris ES Eastell R Reid IR Trial F. Denosumab for prevention of fractures in postmenopausal women with osteoporosis. N Engl J Med. (2009) 361:756–65. 10.1056/NEJMoa0809493 19671655

[56] Inderjeeth CA Chan K Kwan K Lai M. Time to onset of efficacy in fracture reduction with current anti-osteoporosis treatments. J Bone Miner Metab. (2012) 30:493–503. 10.1007/s00774-012-0349-1 22643863

[57] Reynolds K Muntner P Cheetham TC Harrison TN Morisky DE Silverman S Primary non-adherence to bisphosphonates in an integrated healthcare setting. Osteoporos Int. (2013) 24:2509–17. 10.1007/s00198-013-2326-5 23595561

[58] Miller PD Hattersley G Riis BJ Williams GC Lau E Russo LA Effect of abaloparatide vs. placebo on new vertebral fractures in postmenopausal women with osteoporosis: a randomized clinical trial. JAMA. (2016) 316:722–33. 10.1001/jama.2016.11136 27533157

[59] Beckmann J Ferguson SJ Gebauer M Luering C Gasser B Heini P. Femoroplasty—augmentation of the proximal femur with a composite bone cement—feasibility, biomechanical properties and osteosynthesis potential. Med Eng Phys. (2007) 29:755–64. 10.1016/j.medengphy.2006.08.006 17023189

[60] Beckmann J Springorum R Vettorazzi E Bachmeier S Lüring C Tingart M Fracture prevention by femoroplasty—cement augmentation of the proximal femur. J Orthop Res. (2011) 29:1753–8. 10.1002/jor.21410 21500251

[61] Sutter EG Mears SC Belkoff SM. A biomechanical evaluation of femoroplasty under simulated fall conditions. J Orthop Trauma. (2010) 24:95–9. 10.1097/BOT.0b013e3181b5c0c6 20101133 PMC2813465

[62] Fung A Fleps I Cripton PA Guy P Ferguson SJ Helgason B. Prophylactic augmentation implants in the proximal femur for hip fracture prevention: an in silico investigation of simulated sideways fall impacts. J Mech Behav Biomed Mater. (2022) 126:104957. 10.1016/j.jmbbm.2021.104957 34861519

[63] Szpalski M Gunzburg R Aebi M Delimoge C Graf N Eberle S A new approach to prevent contralateral hip fracture: evaluation of the effectiveness of a fracture preventing implant. Clin Biomech. (2015) 30:713–9. 10.1016/j.clinbiomech.2015.05.001 26043935

[64] Springorum HR Gebauer M Mehrl A Stark O Craiovan B Püschel K Fracture prevention by prophylactic femoroplasty of the proximal femur—metallic compared with cemented augmentation. J Orthop Trauma. (2014) 28:403–9. 10.1097/BOT.0000000000000035 24949955

[65] Basafa E Murphy RJ Otake Y Kutzer MD Belkoff SM Mears SC Subject-specific planning of femoroplasty: an experimental verification study. J Biomech. (2015) 48:59–64. 10.1016/j.jbiomech.2014.11.002 25468663 PMC4274194

[66] de Bakker P. Hip fractures: understanding the mechanism and seeking prevention through prophylatic augmentation of the proximal femur (MSc thesis). University of British Columbia, BC, Canada (2006).

[67] Fliri L Sermon A Wähnert D Schmoelz W Blauth M Windolf M. Limited V-shaped cement augmentation of the proximal femur to prevent secondary hip fractures. J Biomater Appl. (2013) 28:136–43. 10.1177/0885328212443274 22492197

[68] Kok J Širka A Grassi L Raina DB Tarasevičius S Tägil M Fracture strength of the proximal femur injected with a calcium sulfate/hydroxyapatite bone substitute. Clin Biomech. (2019) 63:172–8. 10.1016/j.clinbiomech.2019.03.008 30903873

[69] Heini PF Franz T Fankhauser C Gasser B Ganz R. Femoroplasty-augmentation of mechanical properties in the osteoporotic proximal femur: a biomechanical investigation of PMMA reinforcement in cadaver bones. Clin Biomech. (2004) 19:506–12. 10.1016/j.clinbiomech.2004.01.014 15182986

[70] Guido D Raspanti F Gabbiani N Innocenti M Civinini R. Osteo-enhancement procedures in hip fracture prevention: definition and local interventions. Int J Bone Frag. (2022) 2:16–9. 10.57582/IJBF.220201.016

[71] Varga P Hofmann-Fliri L Blauth M Windolf M. Prophylactic augmentation of the osteoporotic proximal femur-mission impossible? Bonekey Rep. (2016) 5:854. 10.1038/bonekey.2016.86 28018586 PMC5141600

[72] Alnemer MS Kotliar KE Neuhaus V Pape HC Ciritsis BD. Cost-effectiveness analysis of surgical proximal femur fracture prevention in elderly: a Markov cohort simulation model. Cost Eff Resour Alloc. (2023) 21:77. 10.1186/s12962-023-00482-4 37880692 PMC10601292

